# Near-Unity All-Optical Modulation of Third-Harmonic Generation with a Fano-Resonant Dielectric Metasurface

**DOI:** 10.1021/acs.nanolett.4c03536

**Published:** 2024-10-02

**Authors:** Falco Bijloo, Kevin Murzyn, Floor van Emmerik, Arie J. den Boef, Peter M. Kraus, A. Femius Koenderink

**Affiliations:** † Advanced Research Center for Nanolithography, Science Park 106, 1098 XG Amsterdam, The Netherlands; ‡ Department of Physics of Information in Matter and Center for Nanophotonics, NWO-I Institute AMOLF, Science Park 104, 1098 XG Amsterdam, The Netherlands; ¶ Department of Physics and Astronomy, and LaserLaB, Vrije Universiteit, 1081 HV Amsterdam, The Netherlands; § ASML Netherlands B.V., 5504 DR Veldhoven, The Netherlands

**Keywords:** Metasurface, All-optical modulation, Third-harmonic generation, Fano resonance

## Abstract

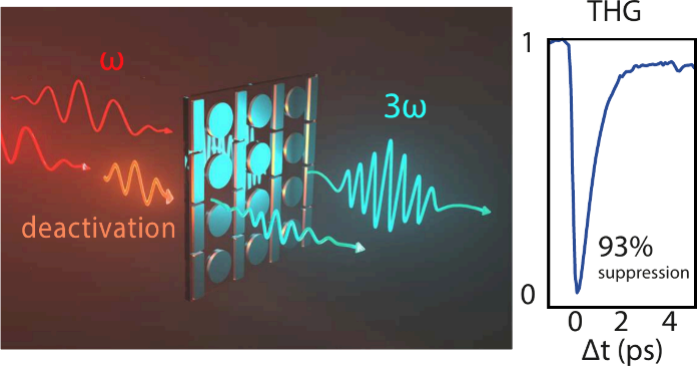

We demonstrate all-optical modulation with a near-unity contrast of nonlinear light generation in a dielectric metasurface. We study third-harmonic generation from silicon Fano-resonant metasurfaces excited by femtosecond pulses at 1480 nm wavelength. We modulate the metasurface resonance by free carrier excitation induced by absorption of an 800 nm pump pulse, leading to up to 93% suppression of third-harmonic generation. Modulation and recovery occur on (sub)picosecond time scales. According to the Drude model, the pump-induced refractive index change blue-shifts the metasurface resonance away from the generation pulse, causing a strong modulation of third-harmonic conversion efficiency. The principle holds great promise for spatiotemporal programmability of nonlinear light generation.

In recent years a strong interest has emerged in all-dielectric optical metasurfaces.^1^ Metasurfaces are nanopatterned 2D systems of strongly scattering meta-atoms arranged at subwavelength spacings, each tailored in their response through shape, size, and orientation. This provides the capability to manipulate amplitude, phase, and polarization of light passing through metasurfaces in completely new ways, leading to applications as flat imaging optics,^2^ augmented reality devices,^3^ holography,^4^ polarimetry,^5^ sensing,^6^ spectroscopy,^7^ and optical signal processing.^8^ Nonlinear metasurfaces offer many additional opportunities, for instance in second-, third-, and high-harmonic generation and four-wave mixing (SHG, THG, HHG, and FWM).^9,10^ These opportunities arise through a confluence of unique properties. First, very large conversion efficiencies are possible by engineering strong resonances. Second, since interaction lengths are far below the wavelength scale, phase-matching requirements are absent. Third, complex wavefronts can be imprinted at will on nonlinear output beams by rational design. A potent route to efficient generation is provided by a Fano-resonant metasurface. Several groups demonstrated efficient THG and HHG,^11−13^ nonlinear holography,^14^ THz wave generation,^15^ nonlinear beam steering,^16^ nonlinear imaging,^17^ and EUV beam shaping.^18^

Widespread application of metasurfaces is limited by the fact that function is fixed at fabrication time and, often, the ability to dynamically control function. Removing this limitation is widely recognized as a main objective. For instance, to advance metasurfaces for analog wave-based processing, it is crucial to develop tunable versions that allow high-contrast and ultrafast control over their linear optical responses.^19^ Similarly, when metasurfaces are used for nonlinear generation, it is highly desirable to dynamically control output beam profile, brightness, wavefront, or polarization. Efforts to create dynamically controllable metasurfaces^19,20^ have so far mainly relied on changes in geometry,^21^ changes in the optical environment surrounding the meta-atoms,^22^ or index changes in the meta-atoms themselves.^23−25^ These changes are realized by temperature tuning,^23^ electrical gating,^26^ strain,^21^ phase-change materials,^24^ or switchable substrates.^22^ Recently, with the use of a nonlinear metasurface, interferometric routing into diffraction orders achieved up to 90% modulation efficiency.^27^ Previous studies on all-optical modulation have demonstrated remarkable advancements and have explored the underlying physics of excited carrier dynamics.^28^ Notable contributions include the modulation of Mie-resonant metasurfaces by free-carrier excitation of direct-gap III–V semiconductor meta-atoms^29^ and broadband modulation with dielectric nanoantennas.^30^ Further, significant progress has been made in SHG modulation within single nanoantennas,^31^ as well as in SHG and THG modulation utilizing gold metasurfaces^32^ and spectral modulation with dielectric metasurfaces.^33^ These works collectively underscore the potential of (nonlinear) metasurfaces for revolutionizing optical modulation. A comprehensive review on active optical metasurfaces reports on recent advances in tunability mechanisms and physics of all-optical modulation.^34^ Although other similar experiments achieved impressive modulation depths,^26,35^ picosecond switching times,^12^ and large routing efficiencies^27^ it is an open challenge to reach high-contrast ultrafast modulation with metasurfaces that perform nonlinear light generation.

In this Letter, we demonstrate picosecond all-optical modulation of nonlinear light generation by a silicon-based all-dielectric Fano-resonant metasurface with near-unity contrast. On basis of the observed transient harmonic generation deactivation, we argue that the all-optical control is based on free carrier excitation upon absorption of the pump pulse, which modulates the silicon refractive index, thereby detuning the sharp Fano resonance, as a consequence dramatically changing the near-field enhancement for the nonlinear generation process and thereby the harmonic conversion efficiency. We argue that this mechanism is generally applicable to Fano-resonant semiconductor metasurfaces and provides a route to ultrafast dynamic and spatial control of nonlinear light generation.

The main idea of our experiment is sketched in Figure 1: we excite a sample with “generation pulses” around 1480 nm and study the third-harmonic (TH) signal that it generates in a pump–probe setup, where an 800 nm pump pulse modulates the TH conversion efficiency by detuning the metasurface resonance. We choose a disk-bar metasurface with a Fano resonance near 1480 nm, advantageous for efficient harmonic generation.^12^ This design is not specifically tailored for this work, and we take it as a simple, well-established THG-generating motif to explore the potential of all-optical modulation of THG generation in Fano-resonant metasurfaces. The metasurface (scanning electron micrograph Figure 2a) is fabricated in polycrystalline silicon evaporated (*d* = 135 nm thickness) on fused quartz patterned with e-beam lithography. The sample consists of a square grid (900 nm pitch) of unit cells consisting of 240 nm radius disks adjacent to 800 × 205 nm bars. The Supporting Information reports in detail on nanofabrication and presents schematics of the experimental setups that were used. The narrow Fano resonances in linear transmission reveal a quality factor of around *Q* = 215 (Figure 2b) and are superimposed on the broad dipole resonance of the bar, which is excited by linear input polarization along the bar. A Fano line shape analysis is found in the Supporting Information. In a standard THG setup (90 fs pulses at 1 MHz repetition rate, with spot size radius around *r* = 11 μm, from a LightConversion Orpheus OPA, tuned near the Fano resonance) the sample presents bright THG, as evident from the emission spectrum and power dependence (Figure 2c and d). At modest input fluences (1 mW input power, i.e., 0.1 mJ/cm^2^ per pulse), the THG intensity increases with a power law close to 3, while for larger fluences (above 3 mW) the TH intensity shows a typical saturation behavior. This saturating dependence is commonly ascribed to two-photon and free carrier absorption.^9,36−38^ Compared to bare silicon, the THG spectrum is a factor 10^3^ brighter, owing to the coupled disk-bar resonance (experimental conditions: integration time 400 ms, at 1 MHz repetition rate and 0.33 mJ/cm^2^ fluence).

**Figure 1:**
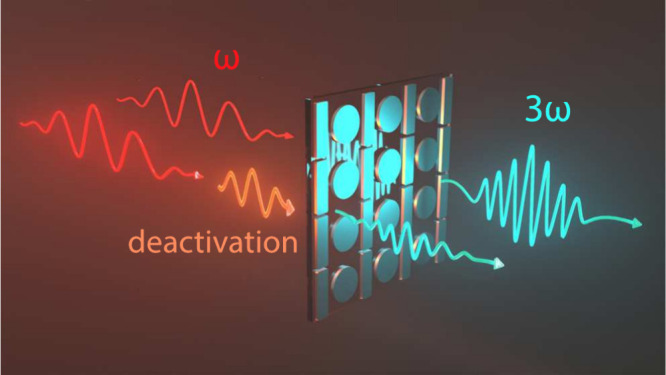
Sketch of our scheme to optically modulate harmonic generation by a metasurface. An IR generation pulse is loosely focused on the Fano-resonant metasurface and converted into a bright third-harmonic signal. Depending on the time delay Δ*t* with respect to the IR excitation pulse, a visible pump pulse can almost fully suppress the harmonic generation.

**Figure 2:**
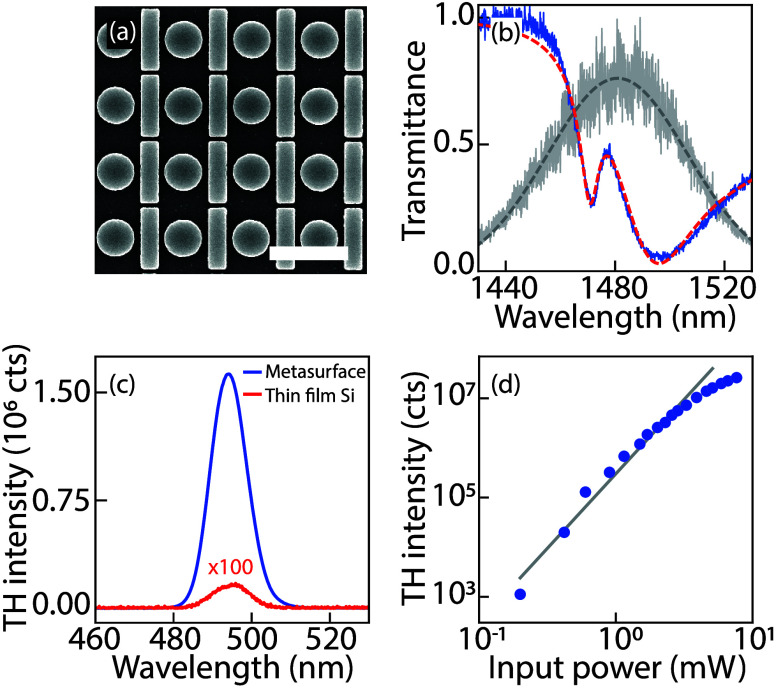
Metasurface properties: (a) scanning electron micrograph of silicon disk-bar metasurface (scale bar 1 μm, pitch 900 nm, disk radius *r* = 240 nm, bar width and length *w* = 205 nm and *l* = 800 nm with gap between disks and bars circa *g* = 50 nm, Si height *d* = 135 nm). (b) Linear transmittance (blue) with a Fano resonance fit (red dashed) showing a quality factor *Q* = 215. For reference the 90 fs generation pulse, used for sample characterization (gray, Gaussian fit dashed), is also shown. (c) TH spectra generated from the metasurface (blue) and unpatterned silicon film (multiplied by 100 for visibility) on the same substrate. (d) Summed TH signal versus input power (blue dots; line shows cubic power law).

The pump–probe setup is based on a Solstice Ace (Spectra-Physics) ultrafast amplifier (2 kHz repetition rate) that amplifies Ti:sapphire laser pulses to millijoule levels. Part of the 800 nm beam feeds into an optical parametric amplifier (TOPAS Prime, LightConversion) that outputs probe pulses at 1480 nm of 51 fs (fwhm) duration (see Supporting Information). The remainder of the 800 nm beam is used as a pump pulse of 72 fs, after passing through an attenuator and delay-stage to control the arrival time relative to the probe pulse. Both pump and probe are loosely focused, with an approximate pump spot size with fwhm 275 μm covering the entire 200 μm metasurface field, and a smaller probe spot with fwhm 62 μm. Half-wave plates independently control the input polarization of both beams. For the remainder of this Letter we refer to the 800 nm pump as “deactivation pulse”, as it suppresses the harmonics generated by the infrared “generation pulse”.

Figure 3 presents the main result of our study, i.e., transient, almost complete suppression of third-harmonic generation. Figure 3a reports the THG spectrum as a function of delay between the generation and deactivation pulse. Negative times correspond to deactivation pulses arriving after the generation pulse, which provides the reference behavior (no deactivation). Just after temporal overlap (maximum suppression occurs at time delay Δ*t* = 100 fs), the THG signal is clearly reduced by approximately 93%, as also quantified in the crosscuts (panel b). This specific measurement is carried out at a pump fluence of 8.5 mJ/cm^2^. The spectra also present a feature at 550 nm, which is due to FWM (occurring at twice 800 nm minus 1480 nm). The FWM signal requires exact temporal pulse overlap owing to the instantaneous nature of the required nonlinearity and indeed occurs only in a single time bin of 100 fs and therefore serves as a yardstick for time Δ*t* = 0. In contrast, the TH suppression relaxes on a slower time scale of a few picoseconds.

**Figure 3:**
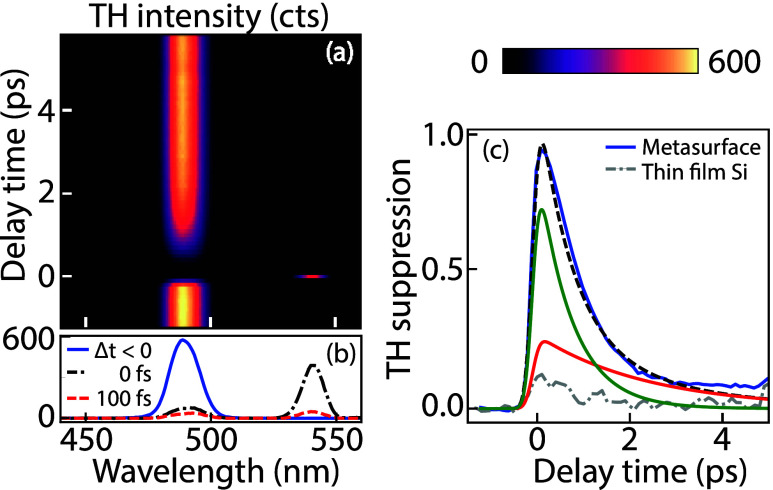
Transient behavior of the THG spectrum, which is exposed to a deactivation pulse (pump fluence 8.5 mJ/cm^2^) at time Δ*t* = 0 ps. (a) TH intensity as a function of pump–probe delay and wavelength. (b) Spectral crosscuts from panel (a) at time Δ*t* < 0 (blue), Δ*t* = 0 fs (black dash-dot), and Δ*t* = 100 fs (red dashed). (c) TH suppression dynamics for the metasurface (blue) and thin film reference (gray dot-dashed), at the same experimental conditions. TH suppression is fitted with a biexponential curve (black dashed) showing two decay mechanisms with a fast (green) and slow decay rate (red).

To quantify the intensity reduction dynamics, we examine the TH suppression as a function of time, plotted in Figure 3c. The TH suppression is defined as *S* = 1 – *I*(Δ*t*)/*I*_0_ where *I*(Δ*t*) refers to the intensity at delay Δ*t* spectrally integrated from 460 to 510 nm and where we normalize to the integrated THG intensity *I*_0_ in absence of the deactivation pulses (at negative Δ*t*). For reference, we also report the transient TH suppression taken at the same experimental settings but on an unpatterned silicon film on the same sample substrate. The very large 93% THG suppression at Δ*t* = 100 fs for the metasurface is at least 7.5 times larger than the 12% suppression observed in the thin film reference. This underlines the important advantage of the resonant mode structure of the metasurface, which is instrumental not only for a large THG efficiency but also for the achievable modulation depth.

We hypothesize that the remarkably strong modulation of THG results from free carriers that are generated by direct absorption of the pump. Indeed, the picosecond time scales of the suppression point at free carrier dynamics. In this picture, the suppression arises because free carriers change the refractive index, detuning the metasurface resonance relative to the probe. Similar spectral shift behavior was observed in refs (28), (29), and (33). The detuning of the resonance results in a strong reduction in THG since the conversion efficiency strongly depends on relative tuning of the metasurface and generation pulse according to experiments on unswitched metasurfaces excited by wavelength-tuned laser pulses.^9,12,13^ Optical switching due to free carrier excitation in silicon was studied extensively in the context of photonic crystals, starting with the work by Leonard et al.^39^ Key expected features are that instantaneous direct pump absorption excites free carriers, which gives rise to a strong change of the silicon refractive index at the generation probe frequency described by the Drude model. Thermalization of electrons on time scales of picoseconds and subsequent carrier recombination on time scales of 10–1000 ps then lead to multiexponential temporal evolution back to the unexcited state. To evaluate this hypothesis, we computed the complex-valued refractive index change as a function of carrier density (Figure 4a) according to the Drude model, which we then used as input for COMSOL finite element calculations of metasurface transmittance. A simplified version of the Drude model, lumping together contributions of electrons and holes, reads^40−42^
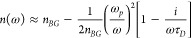
with *n*_*BG*_ = 3.45 the unexcited Si refractive index, τ_*D*_ ≈ 10^–14^ s the relaxation time relevant for polycrystalline Si,^43^ and ω_*p*_ the fluence-dependent plasma frequency ω_*p*_^2^ = *e*^2^*N*/(ϵ_0_*m*_opt_^*^) with *N* the density of electrons plus holes, and *m*_opt_^*^ = (*m*_*e*_^–1^ + *m*_*h*_^–1^) = 0.15*m*_*e*_ the effective carrier mass in units of the mass *m*_*e*_ of the electron.^44^ According to the Drude model, substantial refractive index changes of order Δ*n* = 0.05 occur for carrier densities above *N* > 10^19^ cm^–3^, with the change Δ*n* mainly in the real part as we operate at *ωτ*_*D*_ ≈ 10. The metasurface resonances display a large wavelength sensitivity to refractive index changes, associated with strong local fields in the silicon. At carrier densities on the order of *N* = 10^19^ cm^–3^ already a full line width change of the Fano resonance occurs. At larger excitation density (*N* = 5 × 10^19^ cm^–3^) the index changes cause the resonance to be completely shifted spectrally away from the generation pulse.

**Figure 4:**
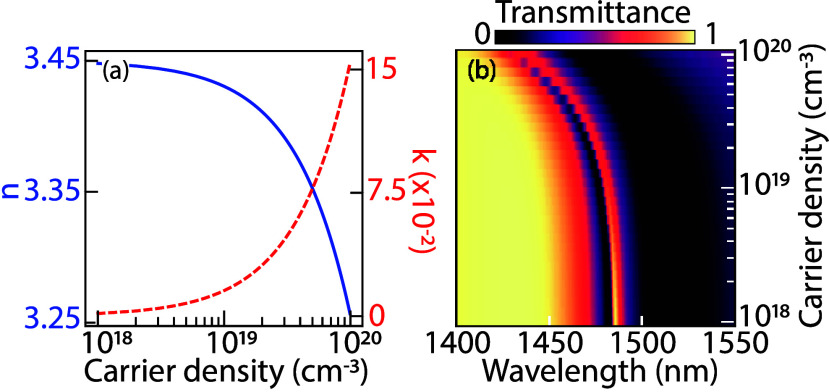
Drude model analysis: (a) calculated real (blue) and imaginary (red dashed) refractive index of poly-Si as a function of carrier density; (b) simulated metasurface transmittance as a function of excited carrier density and wavelength.

To place our experiment in the context of the Drude model, we estimate the generated carrier densities by assessing the absorbed photon flux. We illuminate with 800 nm deactivation pulses of circa Φ ≈ 7 × 10^15^ photons/cm^2^ as estimated for a measured 1 μJ pulse energy and ca. 275 μm spot diameter (see Supporting Information for spot size measurement). COMSOL simulations predict an absorption coefficient *A* on the order of 3.4% at 800 nm, indicating that we reach excited carrier densities of up to *N* ≈ 2Φ*A*/*d* = 10^19^ cm^–3^ (with *d* = 135 nm the Si thickness). Resonance shifts by a full line width are hence fully in range of our experiment. As the THG intensity scales with the third power of the near-field intensity of the generation pulse, even small modulations of the resonance cause a large suppression (e.g., the 93% suppression requires only a 58% change in near-field intensity for the fundamental). In this estimation, we assumed homogeneous distribution of photoexcited carriers and disregarded the spatial overlap with the generation pulse. For further analysis one could look at the effect of a non-homogeneous distribution of excited carriers due to the pump field, spatially overlapping the fundamental field. Our observation of THG suppression can thus be rationalized as a blue-shift of the resonant feature caused by excited carriers, which thereby shifts spectrally away from the generation pulse, causing a strong reduction in the THG conversion efficiency. As we estimate *ωτ*_*D*_ = 10, our model favors the blue-shift over free-carrier absorption as the main contributor.

TH suppression measurements were carried out for pump fluences between 0.2 and 8.5 mJ/cm^2^. Figure 5a shows that the suppression saturates with an increasing pump fluence. A modest 50% suppression is already reached at 1.6 mJ/cm^2^, whereas the extreme 93% suppression is achieved at 8.5 mJ/cm^2^. Higher input fluence resulted in sample damage. To understand the recovery dynamics of the THG as a function of pump fluence, we fitted biexponential transients for all pump fluences and extracted recovery rates. Transient suppression data and fits for all pump fluences are found in the Supporting Information. The suppression dynamics are excellently fitted by a biexponential  with associated fitted decay rates γ_1_ and γ_2_ shown in Figure 5b. We ascribe the fast decay to diffusion of excited carriers from hotspots to the entire resonator, which indeed occurs on the time scale of picoseconds.^29−31^ The slow decay rate is ascribed to carrier recombination. The fitted time scales range from 2 ps to ≫10 ps (exceeding the range of our experiment), slightly faster than values reported in the literature for monocrystalline silicon.^45^ Our metasurface is actually made of polycrystalline silicon, which makes thermalization likely to happen on a faster time scale.^43^ The rates are essentially independent of the pump fluence.

**Figure 5:**
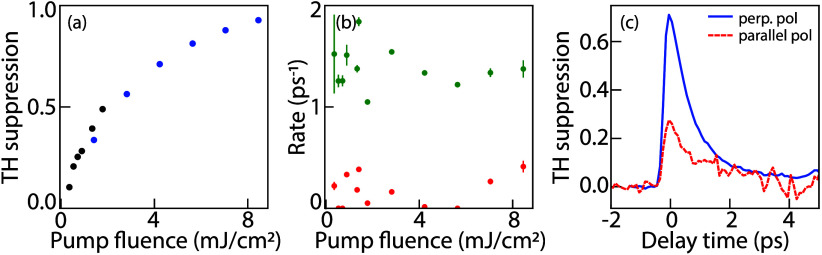
(a) Maximum TH suppression versus deactivation pulse fluence. Different colors (black and blue) correspond to two separate measurement runs. (b) Green (red) symbols: fitted decay rates for biexponential transients versus deactivation pulse fluence for the fast (slow) decay component (c) TH suppression transients at a deactivation pulse fluence of 5.3 mJ/cm^2^, polarized perpendicular (parallel) to the generation pulse in blue (red dashed).

We measured THG suppression for perpendicular and parallel polarizations of the deactivation pulse relative to the metasurface bar (Figure 5c). At perpendicular polarization the suppression is a factor 3 larger compared to the parallel case at identical power. We ascribe this to a different near-field distribution and concomitant difference in absorbed power depending on polarization of the deactivation pulse. COMSOL simulations predict 2.3% absorbed power at 800 nm for parallel polarization versus 3.4% at perpendicular polarization (see Supporting Information). Assuming a homogeneous excited carrier distribution, the absorbed power for 5.3 mJ/cm^2^ for perpendicular polarization is equivalent to (2.3/3.4) × 5.3 = 3.6 mJ/cm^2^ for parallel polarization. When plugging in these numbers in Figure 5a, we expect a reduction of a factor of 2 in TH suppression. The measured factor of 3 might indicate a different spatial distribution of excited carriers at time *t*_0_ and thereby instantaneously a different spatially distributed refractive index. This suggests that the spatial degrees of freedom in free carrier distributions could be used to further control the modulation of THG. In principle, the polarization-dependent TH suppression observation shows that shaping the pump near field provides a route to spatiotemporal control of harmonic generation. Pump field shaping can be achieved by structuring the paraxial incident beam and by engineering the meta-atoms to present spatially varying responses to the pump through, for example, polarization/orientation and local resonance engineering.

In summary, we observed near-unity contrast optically controlled suppression of third-harmonic generation that is generated in an all-dielectric, Fano-resonant silicon metasurface. We explain the suppression by transient detuning of the sharp metasurface resonance relative to the probe pulse, which occurs due to modulation of the complex refractive index of the material through optical excitation of free carriers. These results, combined with the fact that we achieved a very strong modulation strength in an easily fabricated, indirect gap, and unoptimized metasurface, have important implications for a wide range of nonlinear metasurface applications. Nonlinear metasurfaces attract large interest as efficient nonlinear sources of structured light beams, high harmonic beams, and even nonlinear holograms, with applications in free space optics, EUV metrology, microscopy, and integrated optics sources. Our work enables high-contrast, fast dynamical control, and spatial programmability of generated light, greatly advancing the versatility of nonlinear metasurfaces beyond function fixed at nanofabrication. While the suppression achieved here is already remarkably strong, there are many avenues for optimization. Recent work by Koshelev et al. indicates that nonlinear conversion efficiency depends not only on detuning but also on line width matching of probe spectrum and metasurface resonance.^9^ This offers the perspective of control not only via resonance tuning but also via optically induced quality-factor control. Monotonic tuning from high to low *Q* (e.g., by induced free carrier absorption) can lead to both TH enhancement (bringing a system from undercoupling to critical coupling) and TH suppression (into the overcoupled regime). Further, by engineering an absorption resonance at the pump wavelength, we anticipate that the required pump fluences can be reduced by an order of magnitude. Since these effects essentially rely on refractive index tuning at the fundamental wavelength and not on, for example, Kerr-type wave-mixing,^46^ it will also directly apply to high-harmonic generation.^10^ An interesting direction for future research is to apply pump beams that are structured in space or polarization, thereby imprinting dynamically controllable spatial structure in the harmonic suppression or enhancement.^47^ The harmonic generation inherits not only its amplitude but also its local phase pickup from the metasurface. Together, this provides rich opportunities to dynamically control nonlinear generation of structured light,^16,48^ such as orbital angular momentum and vector vortex beams.^49,50^ Finally, in combination with structured illumination our system allows a better understanding of spatiotemporally varying and temporally switched near fields in metasurfaces, accessible via microscopic and interferometric mapping of third-harmonic conversion efficiency.

## Supporting Information Available

Sample fabrication process and resonance characterization; experimental setup and pulse characterization; additional experimental results; simulations of absorbed power simulations (PDF)
