# Supplementary material for: Near-Unity All-Optical Modulation of Third-Harmonic Generation with a Fano-Resonant Dielectric Metasurface
Source: Nano Lett. 2024 Oct 2;24(41):12942–7. doi: 10.1021/acs.nanolett.4c03536 (PMC11487714; doi:10.1021/acs.nanolett.4c03536)
Supplement: Supplementary file 1 — nl4c03536_si_001.pdf [file nl4c03536_si_001.pdf]

Supporting Information:

Near-Unity All-Optical Modulation of Third Harmonic Generation with a Fano-Resonant Dielectric Metasurface

Falco Bijloo,[1,2*] Kevin Murzyn,[1] Floor van Emmerik,[1] Arie J. den Boef,[1,3,4] Peter M. Kraus,[1] and A. Femius Koenderink[2*]

[1]Advanced Research Center for Nanolithography,

Science Park 106, 1098 XG Amsterdam, The Netherlands

[2]Department of Physics of Information in Matter and Center for Nanophotonics,

NWO-I Institute AMOLF, Science Park 104, 1098 XG Amsterdam, The Netherlands

[3]Department of Physics and Astronomy, and LaserLaB,

Vrije Universiteit, 1081 HV Amsterdam, The Netherlands

[4]ASML Netherlands B.V., 5504 DR Veldhoven, The Netherlands

[*]To whom correspondence should be addressed; E-mail: bijloo@amolf.nl; koenderink@amolf.nl

(Dated: September 18, 2024)

I. SAMPLE FABRICATION AND RESONANCE CHARACTERIZATION

A standard e-beam lithography recipe was used to fabricate the nanostructures. First, fused quartz substrates of 12x12 mm and 500 μm thick (Siegert Wafer GmbH) were cleaned by sonication in H_2O for 10 minutes, followed by a dip in a base piranha solution at 75 ˚C for 15 minutes and afterwards rinsed with water and isopropanol (IPA). A layer of 135 nm polycrystalline silicon was evaporated by e-beam evaporation (Polyteknik Flextura M508 E) heating silicon pellets with an emission current of 90 mA to generate a deposition rate of 0.1 nm/s. Next, the sample is put in an oxygen plasma for 2 minutes to grow a thin passivation layer to protect the silicon during development later and to promote adhesive capabilities to the resist layer. Then, a Hydrogen silesquioxane (HSQ) resist layer (Dow Corning, XR-1541 E-Beam Resist) of ca. 65 nm is spincoated with 3000 rpm, 1000 rpm/s, 45s, and baked at 180 ˚C for 2 minutes. To avoid charging during patterning, a thin conductive layer of Elektra (All Resist GmbH, Electra 92) spincoated, 2000 rpm, 1000 rpm/s, 60 s, on top of the HSQ and baked for 2 minutes at 90 ˚C. Patterning is done by exposure to an electron beam in Raith's Voyager system, using an average dose of 1500 $\mu C/cm^2$ (50 kV). The development consists of a dip in water for 15 seconds to remove the elektra layer, 70 seconds in TMAH at 60 ˚C, followed by a rinse of water and IPA for 15 seconds each. The last step is to transfer the HSQ mask in the silicon, which we did via reactive ion etching (Oxford Instruments, Plasma Technologies Plasmalab 80 Plus) using a chemistry of CHF3/SF6/O2 (15/10/3 sccm, Forward power 150W, chamber pressure 7 mTorr), which etches ca. 45 nm/minute. Excess resist layer is not removed, as this did not change the optical properties or impact the quality of the experiment significantly.

The metasurfaces were designed such to present Fano lineshapes between 1400 and 1550 nm. Fano resonances are a result of the coupling between a broad (bright) and a narrow (dark) resonance. In our case, the broad mode is an electric dipole that is located along the long axis of the bar, whereas the narrow mode is a magnetic mode located inside the disk, pointing out of the plane. To calculate the quality factor Q of the Fano resonance, the following procedure is used. First, we set up an equation for the transmission profile of a set of coupled oscillators

$$T = 1 - \chi \sqrt{\gamma_1}$$

with γ_1 the total loss of the broad resonator, and χ the susceptibility

$$\chi = M \cdot I \;\; 2\sqrt{\gamma_1}\, i\, \omega$$

with I the unity matrix, ω the frequency of the light, and M the coupled oscillator matrix

$$M = \begin{pmatrix} -\omega^2 + i\, f\, \omega\, \gamma_1 + \omega_1^2 & -i\, \kappa_{12} & -i\, \kappa_{13} \\ i\, \kappa_{12} & -\omega^2 + i\, \omega\, \gamma_2 + \omega_2^2 & 0 \\ i\, \kappa_{13} & 0 & -\omega^2 + i\, \omega\, \gamma_3 + \omega_3^2 \end{pmatrix}$$

where f tunes leakage (and f=1 means impedance matched), ω_n the resonance frequency of the nth oscillator, κ_{12} the coupling between the broad and narrow resonator, κ_{13} the coupling between the broad and another broad resonator, and γ_2 and γ_3 the losses of the narrow and second broad resonators respectively. We introduced a third oscillator in our analysis, as our experimental data showed a typical strong asymmetric Fano lineshape, superimposed on a slight asymmetric broader resonance profile. This slight asymmetric broad resonance, we ascribed to the coupling between two broad resonances.

Fitting to experimental data, we found the values

$\lambda_1 = \frac{c}{\omega_1}$	$\lambda_2 = \frac{c}{\omega_2}$	$\lambda_3 = \frac{c}{\omega_3}$	$Q_1 = \frac{\omega_1}{\gamma_1}$	$Q_2 = \frac{\omega_2}{\gamma_2}$	$Q_3 = \frac{\omega_3}{\gamma_3}$	κ_{12}	κ_{13}	f
1502.8 nm	1472.4 nm	1530.5 nm	48.98	215.6	20.81	$(20.22\, \gamma_2)^2$	$(4\, \gamma_3)^2$	1

S1.1: Table with fitted parameter values of the Fano model to the experimental transmission data.

Note that the quality factor Q is calculated as $\frac{\omega}{\gamma}$, as it gives the amount of oscillations for that given frequency. The quality factor Q = 215, stated in the main text, is the Q_2 extracted from this fit.

These values leaded to the following fit to the experimental transmission

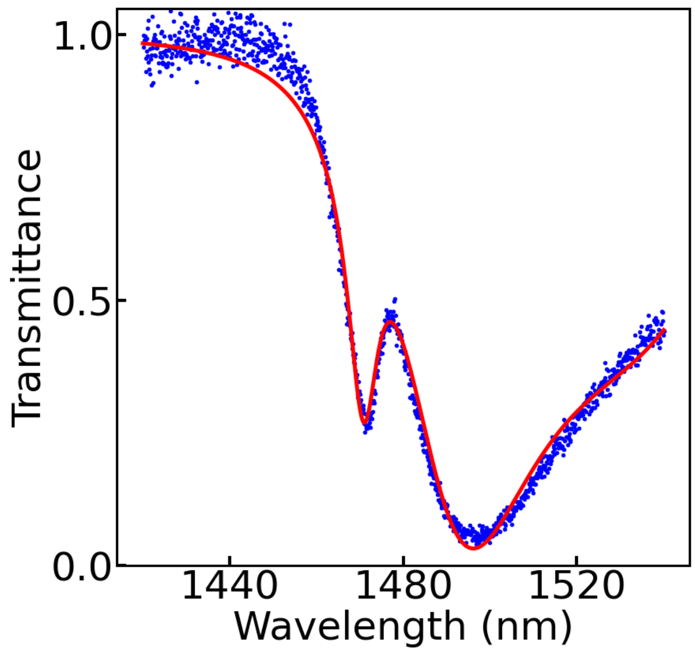

S1.2: Transmittance measurement for the metasurface described in the main text in blue dots, with a coupled oscillator Fano lineshape fit plotted as the red solid line.

II. EXPERIMENTAL SETUPS AND PULSE CHARACTERIZAZTION

Schematics of the setups

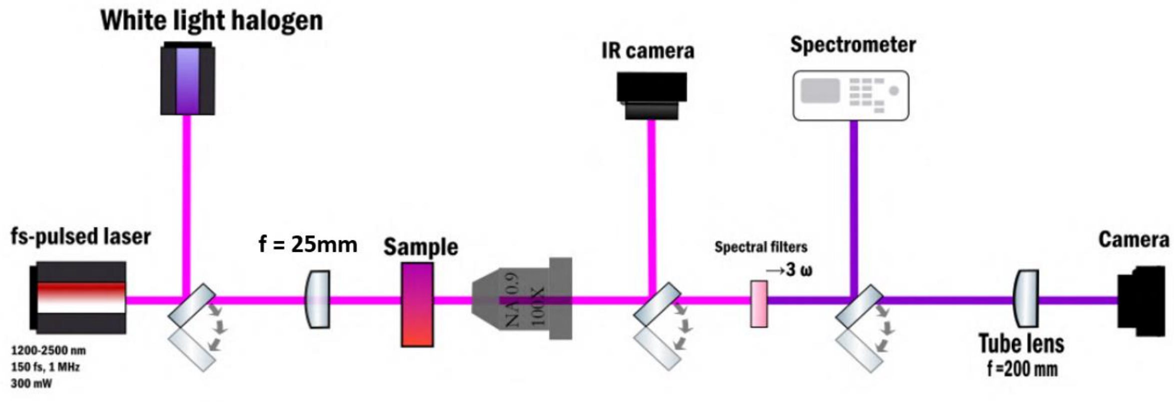

S2.1: Schematic of the experimental setup that is used for sample characterization measurements, shown in Fig. 2.

In the experimental setup for sample characterization measurements, pulses from a femtosecond optical parametric amplifier (idler from OrpheusF, LightConversion) is slightly focused on the sample. A collection objective of NA 0.9 (100x, Nikon, CFI Plan Apo BD) collects all light in transmission. A set of filters (Thorlabs Inc., FESH0650) in combination with a dichroic mirror

(not shown in the schematic, Edmund Optics, Dichroic longpass filter 600nm 69-879) transmits the third harmonic, while getting rid of the infrared light.

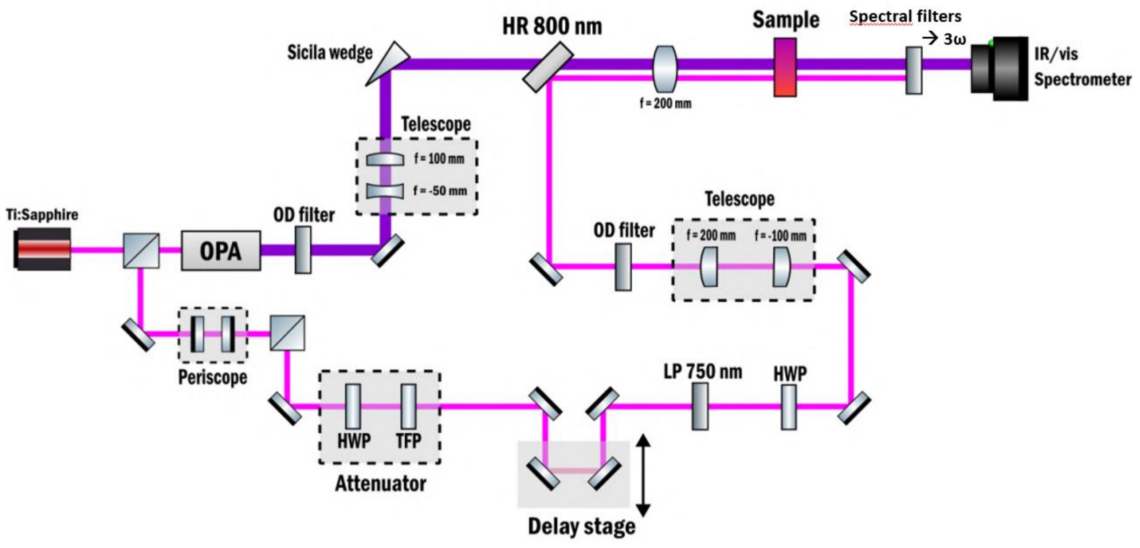

S2.2: Schematic of the experimental setup that is used for transient deactivation measurements, shown in Fig. 3.

The experimental setup used for transient deactivation measurements is based on a modified Mach-Zehnder-interferometer. The top path sends a converted 1480 nm beam through the sample. The bottom path is for the 800 nm beam. The beam passes through a delay stage, allowing for control over the time of arrival with respect to the 1480 nm beam. In addition, a set of HWP and linear polarizer (Eksma, thin-film polarizer) is used as attenuator.

Deactivation pulse characterization

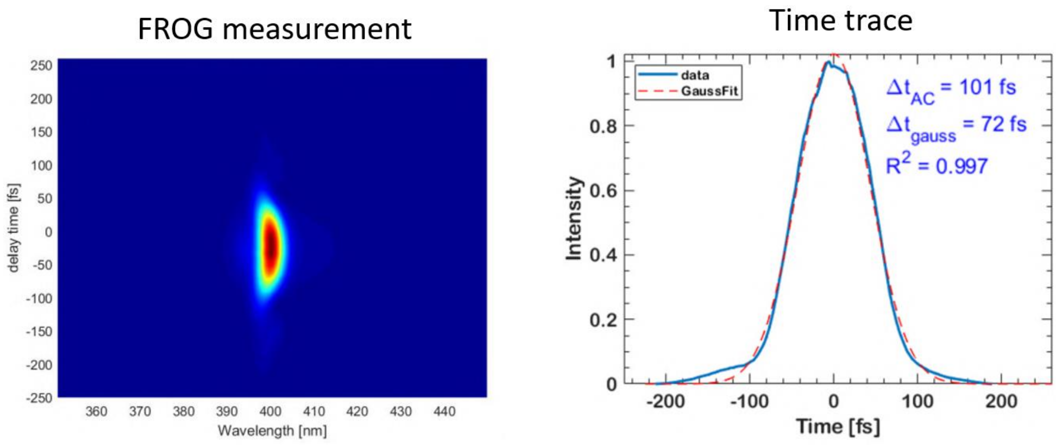

S2.3: FROG and time trace measurements to characterize the deactivation spatial and temporal pulse width.

The wavelength and the duration of the deactivation pulse were measured via frequency-resolved optical gating (FROG) technique. We measured the second harmonic of the pulse, generated in a 20 μm thick β-barium borate crystal (BBO), as this was easier in our setup. Left: reconstructed FROG trace showing intensity as a function of both wavelength and time, right: normalized intensity plotted as a function of time. A Gaussian distribution is fitted to the date, which gives a FWHM of 72 fs.

Beam profile measurement

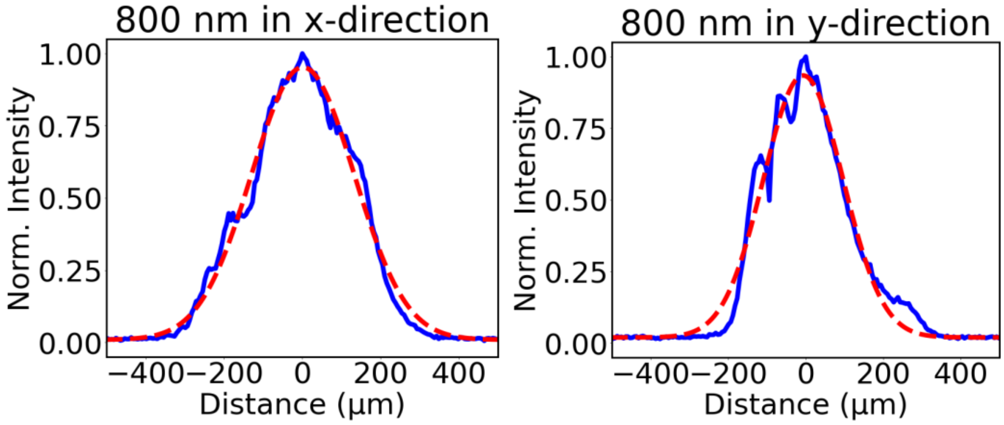

S2.4: Beam profile measurements for the deactivation pulse. Experimental data is plotted as the blue solid line, and the Gaussian fit in red dashed line.

The beam profile was measured for the deactivation pulse at 800 nm. In the above images, Gaussians are fitted with σ_x = 131 μm and σ_y = 102 μm. These correspond to full-width half-maximums in the x-direction of 310 μm and in the y-direction 241 μm. Our estimation of the spot size of 275 μm derives from these values.

Generation pulse spectral measurement

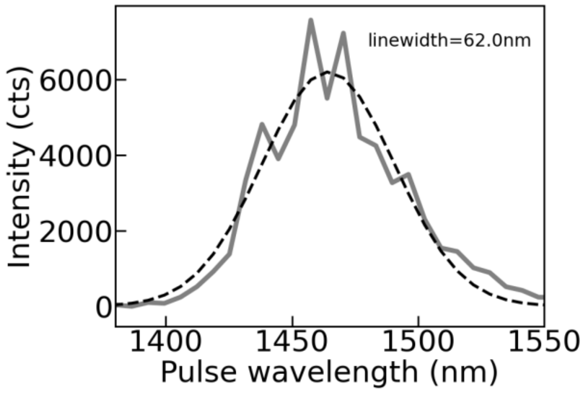

S2.5: Generation pulse spectrum, where the experimental data is plotted as the grey solid line and the Gaussian fit to determine pulse width as the black dashed line.

Also, the spectrum of the generation pulse was measured and a Gaussian with a FWHM = 62 nm was fitted to the spectrum. This relates to a (transform-limited) pulse of 51 fs. The non-Gaussian shape/roughness of the spectrum is an experimental artifact that comes from a problem with either the spectrometer or the fiber leading to the spectrometer.

III. ALL TRANSIENT DATA

This section shows TH suppression traces for each measured pump fluence, with bi-exponential fits that correspond to the datapoints in Fig. 5 in the main text. The pump fluences are given in each transient data plot. From around 4.2 mJ/cm^2 there is a second suppression peak visible around 5 ps delay time. We attribute this to reflection of the deactivation pulse within the substrate. The substrate thickness of 500 µm, translates to ~ 5 ps travel time. The second peak appears to grow more strongly than the 0 ps main suppression peak. We attribute this to the fact that in this regime the main suppression at 0 ps is already in the saturation regime.

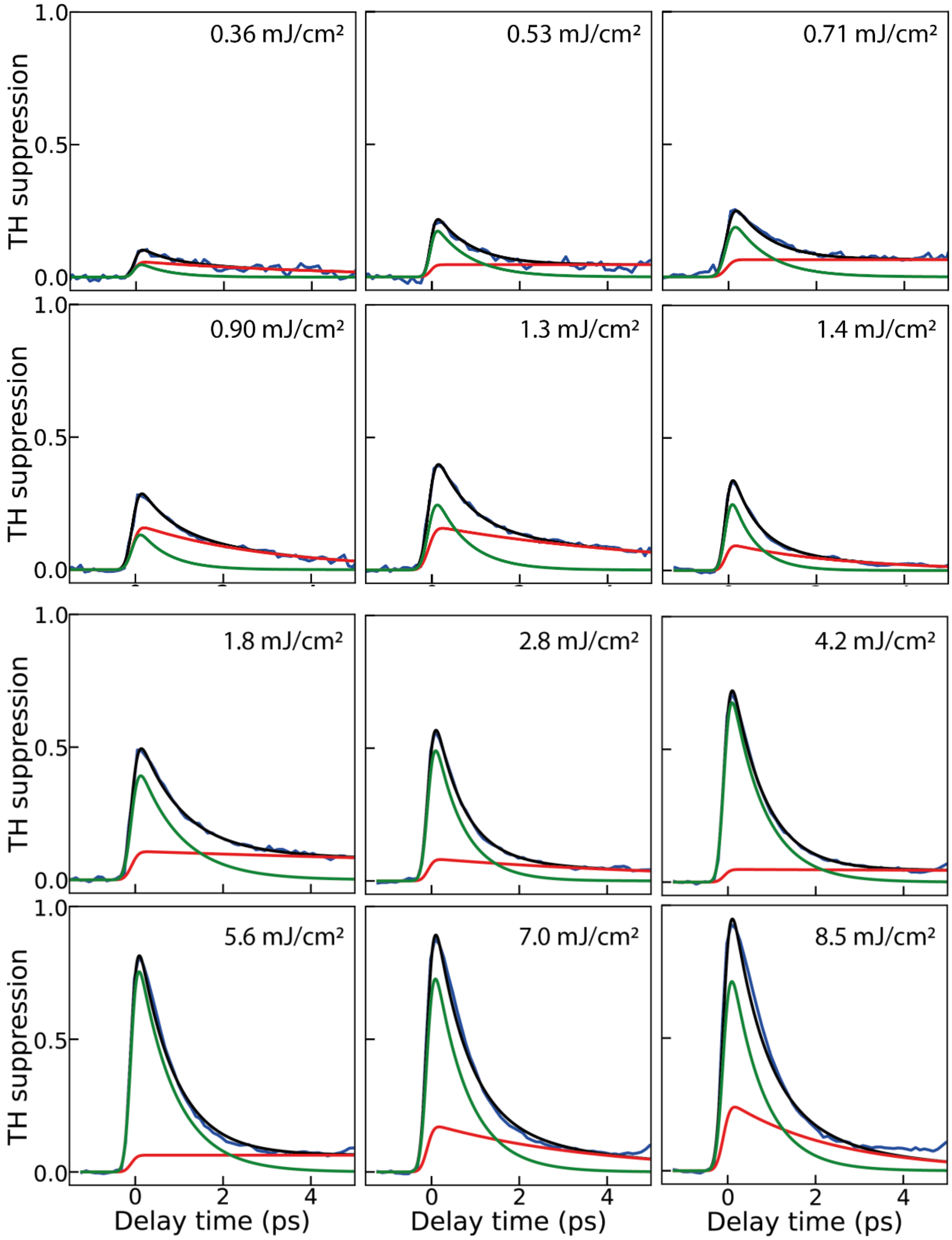

S3.1 Transient TH suppression data for all measured pump fluences. The experimental data is plotted as the blue curve, the black curve presents the bi-exponential fit, whereas the green (red) lines present the fast (slow) decaying exponentials.

In the main text Fig. 5b, we plotted the fitted parameter values to the decay traces as decay rates, as these are directly extracted from the fit, and generally used to describe decay mechanisms. For researchers wishing to assess the decay times associated with the fast component, we also present a decay time vs pump fluence plot.

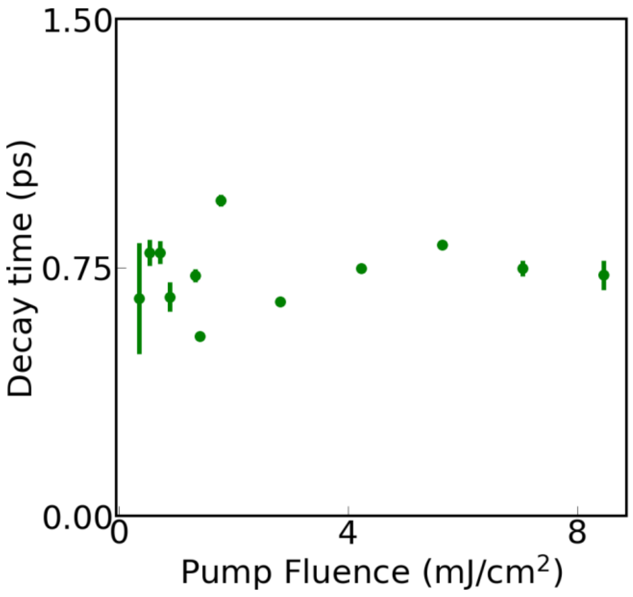

S3.2: Decay time $1/\gamma_2$ as function of pump fluence, as extracted from the fast decay exponential fits in S3.1.

Note that we did not include $1/\gamma_2$ in this plot, as these timescales vary much and are quite larger than $1/\gamma_1$.

IV. ABSORBED POWER SIMULATION FOR DIFFERENT POLARIZATIONS

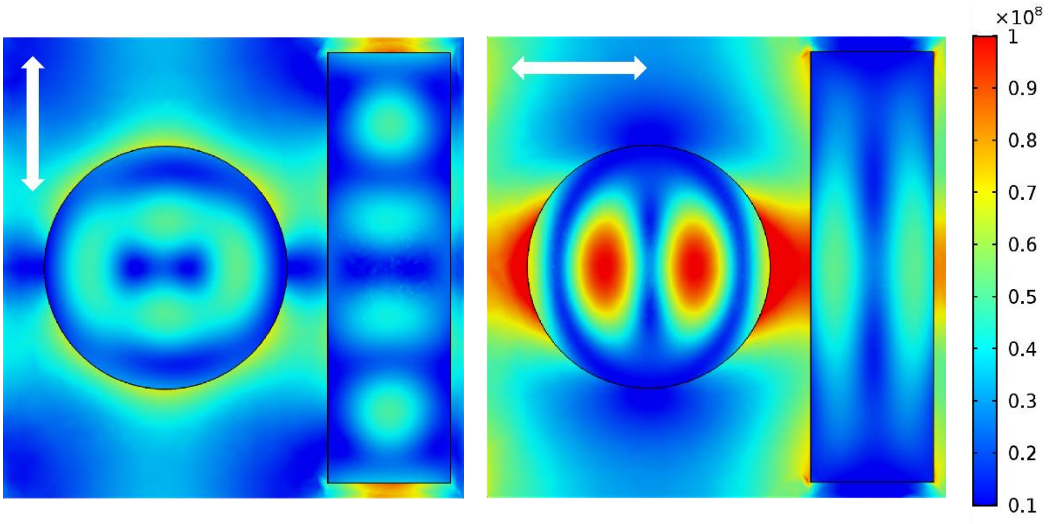

S4.1: COMSOL simulation to calculate absorbed power. One meta-atom is simulated with periodic boundary conditions, and the color bar represents normalized electric field. The polarization of the field is given by the white arrow in the top left corner.

COMSOL simulation to calculate absorbed power. Plotted in these images is the normalized electric near field, both represented with the same color scale, which is plotted on the right. These simulations are performed at a single frequency as opposed to pulsed excitation, with incoming vacuum wavelength set to 800nm. Dimensions are similar as described in the main text. The left (right) image is for vertical (horizontal) polarization. Absorbed power in the disk plus bar are 2.3% for the vertical polarization case and 3.4% for horizontal polarization.
